# Proteomic profiling and comparative hemotoxicity of *Gloydius brevicaudus* and *Deinagkistrodon acutus* venoms: insights into functional convergence

**DOI:** 10.1590/1678-9199-JVATITD-2026-0022

**Published:** 2026-08-28

**Authors:** Jianguo Hu, Hongxia Guo, Nian Wang, Xinghuai Cai

**Affiliations:** 1 Faculty of Modern Health Care, Anhui Sanlian University, Hefei, China.; 2 Student Affairs Office, Anhui Zhong-Ao Institute of Technology, Hefei, China.

**Keywords:** Gloydius brevicaudus, Deinagkistrodon acutus, proteomics, SVMP, SVSP

## Abstract

**Background:**

Snakebite envenomation caused by *G. brevicaudus* and *D. acutus* frequently presents with overlapping hemorrhagic and coagulopathic manifestations in clinical settings. However, whether these similarities reflect convergent venom phenotypes remains unclear.

**Methods:**

We performed an integrated comparative analysis combining nano-LC-MS/MS proteomics with multi-target functional assays to systematically evaluate venom composition and biological activities. Proteomic profiling was used to characterize toxin family abundance, while enzymatic, cytotoxic, hemolytic, and cell signaling assays were conducted to assess functional effects.

**Results:**

Proteomic analysis revealed that both venoms are predominantly composed of hemotoxic toxin families, including snake venom metalloproteinases (SVMP), snake venom serine proteases (SVSP), C-type lectin-like proteins (CTLP), and phospholipase A₂ (PLA₂), with broadly comparable relative abundances. Functional assays demonstrated similar concentration-dependent patterns in proteolytic, PLA₂, thrombin-like, and fibrinolytic activities, with no statistically significant interspecific differences. Both venoms also induced comparable cytotoxic effects across mammalian cell lines, while exhibiting limited hemolytic activity and minimal modulation of Ca²⁺ signaling and nitric oxide production.

**Conclusions:**

These findings demonstrate that *G. brevicaudus* and *D. acutus* venoms share a convergent hemotoxic functional architecture characterized by consistent enzymatic activities and similar cytotoxic profiles. This functional convergence provides a mechanistic basis for clinically overlapping hemorrhagic and coagulopathic manifestations observed in envenomation cases. The results further emphasize the importance of function-oriented venom profiling and suggest potential implications for antivenom cross-reactivity and therapeutic development.

## Background

Snakebite envenomation remains a major public health burden in China, where viperid snakes account for a substantial proportion of clinically significant cases [1-3]. Among them, *G. brevicaudus* and *D. acutus* are medically important species responsible for frequent envenomation incidents across distinct geographic regions [1]. *D. acutus* is mainly distributed in southern China and parts of Southeast Asia, whereas *G. brevicaudus* has a broader distribution across China, the Korean Peninsula, and adjacent regions. Despite differences in ecological distribution, both species share similar feeding habits and seasonal behaviors [2].

Clinically, envenomation by these two species often presents with overlapping manifestations, including hemorrhage, coagulopathy, and local tissue injury, complicating early diagnosis and clinical management [3-8]. However, notable differences in venom yield and toxicity have been reported. Adult *D. acutus* can deliver a substantially higher venom dose (approximately 622 mg crude venom, ~160 mg dried weight), often resulting in severe hemorrhagic syndromes and extensive tissue necrosis [9-12]. In contrast, *G. brevicaudus* produces a lower venom yield (approximately 63.11 mg crude venom, ~19.2 mg dried weight) but is associated with broader systemic manifestations, including neurological symptoms and multi-organ dysfunction [13, 14]. Toxicological studies further indicate that *G. brevicaudus* venom exhibits a lower LD₅₀ (0.49 mg/kg, intraperitoneal) compared with that of *D. acutus* (4.4 mg/kg), suggesting higher intrinsic toxicity [15].

Although these clinical features suggest both overlap and divergence in envenomation syndromes, the molecular basis underlying their shared pathological manifestations remains unclear. In particular, it is unknown whether clinically overlapping symptoms reflect convergence in venom composition and functional activity or arise from distinct toxin-driven mechanisms.

Previous proteomic studies have independently characterized both venoms. *D. acutus* venom is enriched in snake venom metalloproteinases (SVMP), serine proteases (SVSP), C-type lectin-like proteins (CTLP), and phospholipase A₂ (PLA₂), which are strongly associated with hemorrhagic and coagulopathic effects [16-19]. In contrast, *G. brevicaudus* venom contains a broader toxin repertoire, including disintegrins, cysteine-rich secretory proteins (CRISP), and L-amino acid oxidases (LAAO), in addition to its major hemotoxic components [20, 21].

However, these studies were performed using different experimental platforms and analytical workflows, limiting direct comparability and preventing an integrated functional interpretation.

Resolving whether these overlapping clinical manifestations reflect true phenotypic convergence is critical for understanding envenomation mechanisms and improving therapeutic strategies, particularly regarding antivenom cross-reactivity.

To address this limitation, a unified analytical framework is required. Proteomic profiling enables the systematic characterization of toxin composition, whereas functional assays directly evaluate the biologically relevant activities underlying clinical manifestations. The integration of these approaches provides a comprehensive phenotype-level comparison beyond descriptive compositional analysis.

Here, we hypothesize that the clinically overlapping envenomation syndromes induced by *G. brevicaudus* and *D. acutus* are driven by convergent hemotoxic venom phenotypes. To test this hypothesis, we applied an integrated nano-LC-MS/MS-based proteomic and multi-target functional framework to systematically compare the venom composition and biological activities of the two species.

## Methods

### Venom samples

Lyophilized venoms of *G. brevicaudus* and *D. acutus* were obtained from the Snake Research Institute of Huangshan City, Anhui Province, China (batch numbers: GB20230712 and DA20230720, respectively). Venoms were collected by the supplier from adult specimens using the plate-bite method. For each species, venoms were pooled from multiple adult individuals to minimize inter-individual variation and obtain representative venom profiles. Specifically, according to the supplier, the pooled *G. brevicaudus* venom was collected from approximately 100 adult individuals, whereas that of *D. acutus* was obtained from approximately 80 adult individuals. All snakes originated from the Huangshan region of Anhui Province, China. After collection, venoms were lyophilized and stored at −20 °C until further use.

### Nano-LC-MS/MS analysis and protein identification

For proteomic analysis, lyophilized venom samples (100 μg) from each species were reconstituted in 50 mM ammonium bicarbonate buffer (pH 7.8). Proteins were reduced with dithiothreitol, alkylated with iodoacetamide, and digested overnight at 37 °C using sequencing-grade modified trypsin at an enzyme-to-substrate ratio of 1:50 (w/w). Digestions were performed using a single-enzyme strategy. Peptide mixtures were desalted using C18 ZipTip pipette tips (Millipore, Bedford, MA, USA) and analyzed in technical triplicate.

Peptides were dissolved in 0.1% formic acid, and approximately 1 μg of digest was injected. Peptides were first loaded onto a trap column (75 μm × 2 cm, Acclaim PepMap C18, 3 μm, 100 Å; Thermo Fisher Scientific) at 5 μL/min for 5 min using 0.1% formic acid in water, and then separated on a nanoViper C18 analytical column (75 μm × 150 mm, 2 μm, 100 Å; Thermo Fisher Scientific) at 300 nL/min with a 5-35% solvent B gradient (0.1% formic acid in acetonitrile) over 90 min.

The column effluent was analyzed using a Vanquish Neo UHPLC system (Thermo Fisher Scientific, Waltham, MA, USA) coupled to an Orbitrap Exploris 480 mass spectrometer equipped with a nano-electrospray ionization (nano-ESI) source. Ionization was performed in positive mode with a spray voltage of 2.1 kV and an ion transfer tube temperature of 275 °C. All other source parameters were operated under standard nano-ESI settings recommended by the manufacturer.

Full MS scans were acquired over an m/z range of 300-1800 at a resolution of 70,000. Data-dependent MS/MS acquisition was performed for the top 10 most intense precursor ions with higher-energy collisional dissociation (HCD) at a normalized collision energy of 35%. Dynamic exclusion was set to 30 s with a mass tolerance of ± 10 ppm.

Raw data were processed using MaxQuant (Andromeda search engine) against the UniProtKB/TrEMBL Serpentes database (release 2023_05). Search parameters included a precursor mass tolerance of 30 ppm and fragment mass tolerance of 0.15 Da. Carbamidomethylation of cysteine was set as fixed modification, whereas N-terminal acetylation, deamidation (N/Q), and oxidation (M) were designated as variable modifications. Trypsin was specified as the protease with up to two missed cleavages. The false discovery rate (FDR) was set to 1% at the peptide and protein levels. Only proteins identified with at least one unique peptide at ≥ 95% confidence were retained.

Relative protein abundance was estimated using intensity-based absolute quantification (iBAQ). For comparative analysis, proteins were assigned to toxin families based on curated annotations, and family abundances were normalized to total venom iBAQ intensity. Toxin families with fewer than three protein entries were grouped as “Others” and excluded from functional interpretation.

### Reverse-phase high-performance liquid chromatography (RP-HPLC)

RP-HPLC was performed as an orthogonal qualitative approach to visualize the overall venom protein complexity and hydrophobicity distribution. Lyophilized venom (100 µg) was dissolved in double-distilled water containing 0.1% trifluoroacetic acid (TFA; T103294, Aladdin Biochemical Technology Co., Ltd., Shanghai, China) and analyzed using a Vanquish Core HPLC system (Thermo Fisher Scientific, Waltham, MA, USA) equipped with a Shimsen Ankylo C18 column (3 µm, 50 × 4.6 mm). Separation was conducted at a flow rate of 2 mL/min using a linear gradient of solvent B (acetonitrile with 0.1% TFA): 0-5 min, 0%; 5-8 min, 0-15%; 8-23 min, 15-45%; 23-26 min, 45-70%; 26-30 min, 70%. Elution was monitored at 280 nm using a diode array detector. Blank runs were used for baseline correction.

### SDS-PAGE

Electrophoretic analysis was conducted to evaluate the overall protein composition of the two venoms. Lyophilized venom samples (20 µg per lane) were reconstituted in loading buffer (P0015, Beyotime Biotechnology, Shanghai, China), heated in boiling water for 5 min, and separated on 4-20% SDS-PAGE precast gels (P0057A, Beyotime Biotechnology, Shanghai, China) using a VE180 vertical electrophoresis unit (Tanon, Shanghai, China). Electrophoresis was conducted at 80 V for 20 min, followed by 120 V until the dye front reached the gel bottom. A prestained molecular weight marker (10-150 kDa; P0060M, Beyotime Biotechnology, Shanghai, China) was used as a reference. Gels were stained with Coomassie Brilliant Blue (P0003S, Beyotime Biotechnology, Shanghai, China), destained, and imaged using a Tanon 1600 gel documentation system.

### Protein concentration determination

Protein concentrations were determined using a Modified Lowry Protein Assay Kit (P0401S, Beyotime Biotechnology, Shanghai, China) according to the manufacturer’s instructions. Bovine serum albumin was used to generate a standard calibration curve (0-1.5 mg/mL). Absorbance was measured at 660 nm using a microplate reader, and all measurements were performed in triplicate.

### Bioactivity assays

All bioactivity assays were performed using lyophilized venom samples. Venom concentrations for each assay were selected based on literature precedents and preliminary range-finding experiments. Cytotoxicity was evaluated using three mammalian cell lines (MDCK II, HEK 293T, and RAW 264.7), and hemolytic activity was assessed using erythrocytes from mice, sheep, and chickens. All experiments were performed in three independent biological replicates (n = 3).

*Cytotoxicity assay*

The cytotoxic effects of the two venoms were evaluated using three mammalian cell lines representing distinct physiological systems potentially involved in envenomation. These cell lines were selected to model different biological processes relevant to venom-induced pathology, including epithelial integrity, immune and inflammatory responses, and mammalian cellular signaling. The use of multiple cell models enabled broader evaluation of potential similarities and differences in venom-induced cytotoxic phenotypes between *G. brevicaudus* and *D. acutus*.

Madin-Darby canine kidney II (MDCK II) cells (FH0896, Fuheng Biology, China) were used as a model for epithelial and renal-derived tissues relevant to organ injury and systemic toxicity. Murine macrophages (RAW 264.7) (CL-0190, Procell Life Science & Technology Co., Ltd., Wuhan, China) were included to assess immune-related responses, as macrophages play a central role in inflammation and venom-induced immunomodulation. Human embryonic kidney cells (HEK 293T) (CL-0005, Procell Life Science & Technology Co., Ltd., Wuhan, China) were used to provide a human-derived cellular context for evaluating venom-induced cytotoxicity and cellular signaling responses [22].

This multi-cell-line strategy was employed to determine whether clinically overlapping envenomation syndromes are associated with convergent cytotoxic profiles across epithelial, immune, and human-derived cellular systems, thereby supporting the central hypothesis of this study.

Cells were cultured in high-glucose DMEM (PM150234, Procell Life Science & Technology Co., Ltd., Wuhan, China) supplemented with 10% fetal calf serum (FCS) and 1% penicillin-streptomycin (C0222, Beyotime Biotechnology, Shanghai, China) at 37 °C in a humidified 5% CO₂ incubator. Cells were seeded in appropriate plates and allowed to adhere prior to treatment. Venoms were applied at three concentrations (25, 2.5, and 0.25 µg/mL). ddH₂O and ionomycin (100 µM) were used as negative and positive controls, respectively. After 48 h of incubation, cell viability was determined using the CellTiter-Glo® luminescent assay (G7570, Promega, Madison, WI, USA) according to the manufacturer’s instructions. Luminescence was recorded using a EnSight Multimode Plate Reader (PerkinElmer, Waltham, MA, USA). Cell viability was expressed as a percentage relative to the negative control group (100%).

*Protease activity assay*

Total protease activity was measured using the Folin-phenol method. Venom samples (100, 50 and 25 µg/mL) were incubated with 2% casein (C755725, Aladdin Biochemical Technology Co., Ltd., Shanghai, China) in 0.2 M Tris-HCl buffer (pH 8.5) at 37 °C for 2 h. The reaction was terminated by adding 1 mL of 0.44 M trichloroacetic acid (TCA), followed by incubation at 37 °C for 30 min and centrifugation at 12,000 × g for 15 min. Supernatants (0.8 mL) were mixed with 2.0 mL of 0.4 M Na₂CO₃ and 0.4 mL of Folin’s reagent, incubated in the dark for 20 min, and the absorbance was measured at 660 nm. While ddH₂O served as the negative control, trypsin (170 µg/mL) served as the positive control. Activity was normalized to 100% of the positive control. 

*PLA₂ activity*

Following a modified protocol by Memar et al. [23], venom samples (50, 25 and 12.5 µg/mL) were mixed with substrate buffer containing 0.1 M NaCl, 10 mM CaCl₂, 7 mM Triton X-100, 0.35% soybean lecithin, and 9.88 mM phenol red (pH 7.6). After a 5 min incubation at room temperature, the absorbance was measured at 550 nm. Purified *Crotalus adamanteus* venom PLA₂ (15 U/mL; P128568, Aladdin Biochemical Technology Co., Ltd., Shanghai, China) was used as the positive control, and the assay buffer served as the negative control. Results were normalized to 100% of the positive control.

*Hemolytic activity*

Hemolytic activity was assessed to evaluate the ability of venoms to disrupt erythrocyte membranes, which represents a key mechanism underlying hemotoxic effects during envenomation. The assay was modified from the method described by Sæbø et al. **[** 24 **]**.

Erythrocytes from murine, ovine, avian (Shanghai Yuanmu Biotechnology Co., Ltd, China), and human (Huizhi Heyuan Biotechnology (Suzhou) Co., Ltd, China) sources were used to enable comparative evaluation of venom-induced hemolytic activity across different vertebrate erythrocyte models. This multi-species design supports the central aim of the study by facilitating functional comparison of the hemotoxic profiles of *G. brevicaudus* and *D. acutus* venoms.

Venoms from both species (5-80 µg/mL) were incubated with 1% erythrocyte suspensions in 96-well V-bottom plates. Triton X-100 (1%) and Alsever’s buffer were used as positive and negative controls, respectively. Samples were incubated at 37 °C with shaking at 130 rpm for 60 min, followed by centrifugation at 804 × g for 5 min at 4 °C. The absorbance of the supernatant was measured at 405 nm using a microplate reader. Hemolysis was expressed as a percentage relative to the positive (100%) and negative (0%) controls. 

*Fibrinolytic activity*

Fibrinolytic activity was determined according to the method of Avella et al. [19] using a Sigma Fibrinolytic Activity Assay kit (MAK244, Sigma-Aldrich). Reaction mixtures contained 2 µL of substrate, 48 µL of buffer, and different venom concentrations (50, 100, and 200 µg/mL) or a positive control (5 µg/mL plasmin). After 55 min of incubation at 37 °C in the dark, fluorescence was measured using a PerkinElmer EnSight Reader (excitation λ = 360 nm, emission λ = 450 nm). Activities were normalized to 100% of the positive control.

*Thrombin activity*

Thrombin activity was determined using a fluorometric thrombin assay kit (MAK242, Sigma-Aldrich) according to the method of Avella et al. [19]. Each well contained 5 µL of substrate, 45 µL of reaction buffer, and various venom concentrations (12.5, 25, and 50 µg/mL) or a thrombin standard (0.3 µg/mL). Following incubation at 37 °C for 55 min in the dark, fluorescence was measured at λ ex = 350 nm and λ em = 450 nm. Results were normalized to the positive control.

*Intracellular Ca²⁺ assay*

Intracellular Ca²⁺ release was quantified according to the method of Erkoc et al. [25]. HEK 293T and MDCK II cells (2 × 10⁴ cells/well) were seeded on poly-D-lysine-coated 96-well plates and incubated for 24 h at 37 °C. Cells were loaded with 4.19 µg/mL Fluo-8 AM (21080, AAT Bioquest, USA) in Hank’s balanced salt solution (HBSS) for 1h, then washed and maintained in 100 µL HBSS. Images were acquired using an ImageXpress Micro Confocal System (Molecular Devices, Germany) at 5 frames/s. For induction assays, cells were exposed to venoms (12.5, 7.5, 2.5, and 0.25 µg/mL), ionomycin (5 µM, positive control), or ddH₂O (negative control) and imaged at 1 frame/s for 20 s. For inhibition assays, cells were pretreated with venoms for 30 min before ionomycin addition. Fluorescence data were analyzed using MetaXpress software, with signal thresholding to count activated cells. Data were normalized to the positive or negative controls (100%).

*Nitric oxide (NO) assay*

The effect of venoms on NO production was quantified in RAW 264.7 macrophages according to a modified protocol by Erkoc et al. [25]. Cells (2 × 10⁴ per well) were seeded in 96-well plates and incubated for 24 h at 37 °C. For induction assays, cells were treated with venoms (25, 2.5, and 0.25 µg/mL), ddH₂O (negative control), or lipopolysaccharide (LPS, 0.1 µg/mL; positive control) to stimulate NO synthesis. For inhibition assays, cells were pretreated with venoms or H₂O₂ for 30 min before LPS addition. After 24 h, 80 µL supernatant was mixed with 20 µL sulfanilamide (40 mg/mL in 1 M HCl) and 20 µL naphthylethylenediamine (60 mg/mL in water), incubated for 15 min, and the absorbance was measured at 540 nm. Data were normalized to the positive (0%) or negative (100%) control values.

### Statistical analysis

Statistical analyses were performed using IBM SPSS Statistics 27. All comparisons between the two venoms (*G. brevicaudus* and *D. acutus*) were performed using Student’s *t*-test (two-tailed), a parametric test appropriate for comparing two independent groups. Given the limited sample size and common practice in venom-related biochemical studies, data were treated as approximately normally distributed. Results are presented as mean ± standard deviation, and differences were considered statistically significant at *p* < 0.05. All experiments were performed in triplicate.

## Results

### Comparative venom proteomic analysis using an integrated analytical approach

Venom protein composition was comprehensively characterized by integrating nano-LC-MS/MS-based proteomics, conventional SDS-PAGE profiling, and RP-HPLC fractionation followed by SDS-PAGE analysis of individual fractions.

*Proteomic comparison of venom composition using nano-LC-MS/MS*

**Figure 1.**
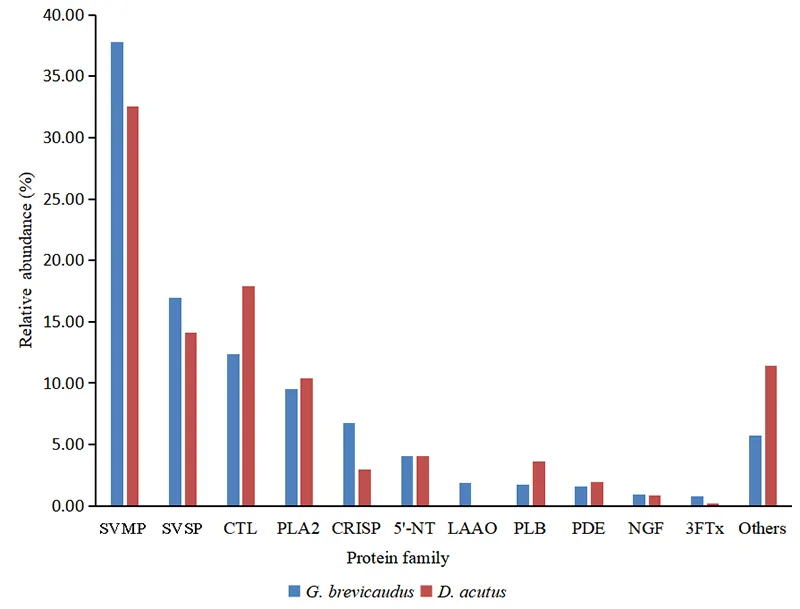
Comparative relative abundance of venom protein families identified in *G. brevicaudus* and *D. acutus* venoms based on normalized abundance values obtained from MaxQuant iBAQ values. 3FTx: three-finger toxin; 5′-NT: 5′-nucleotidase; CRISP: cysteine-rich secretory protein; CTLP: C-type lectin-like protein; LAAO: L-amino acid oxidase; NGF: nerve growth factor; PDE: phosphodiesterase; PLA_2_: phospholipase A_2_; PLB: phospholipase B; SVMP: snake venom metalloproteinase; SVSP: snake venom serine protease.

Proteomic analysis identified 259 protein entries in *G. brevicaudus* venom (Additional file 1A) and 197 protein entries in *D. acutus* venom (Additional file 1B). Among these, 147 protein entries were shared between the two venoms, accounting for 56.76% of the total protein entries identified in *G. brevicaudus* and 74.62% of those identified in *D. acutus*. Furthermore, 11 toxin families with more than three identified protein entries were detected in *G. brevicaudus* venom, including snake venom metalloproteinase (SVMP), snake venom serine protease (SVSP), C-type lectin-like protein (CTLP), phospholipase A₂ (PLA₂), cysteine-rich secretory protein (CRISP), 5′-nucleotidase (5′-NT), L-amino acid oxidase (LAAO), phospholipase B (PLB), phosphodiesterase (PDE), nerve growth factor (NGF), and three-finger toxin (3FTx). In *D. acutus* venom, ten toxin families with more than three identified protein entries were detected, including SVMP, SVSP, CTLP, PLA_2_, CRISP, 5′-NT, PLB, PDE, NGF, and 3FTx, whereas the LAAO family was undetected. The dominant toxin families were consistent between the two venoms (SVMP, SVSP, CTLP, and PLA_2_), with SVMP representing the most abundant family in both species (Figure 1). Collectively, these four major toxin families accounted for 76.62% of the total venom abundance in *G. brevicaudus* and 74.94% in *D. acutus* (Additional file 2).

*RP-HPLC chromatographic comparison of the two venoms*

**Figure 2.**
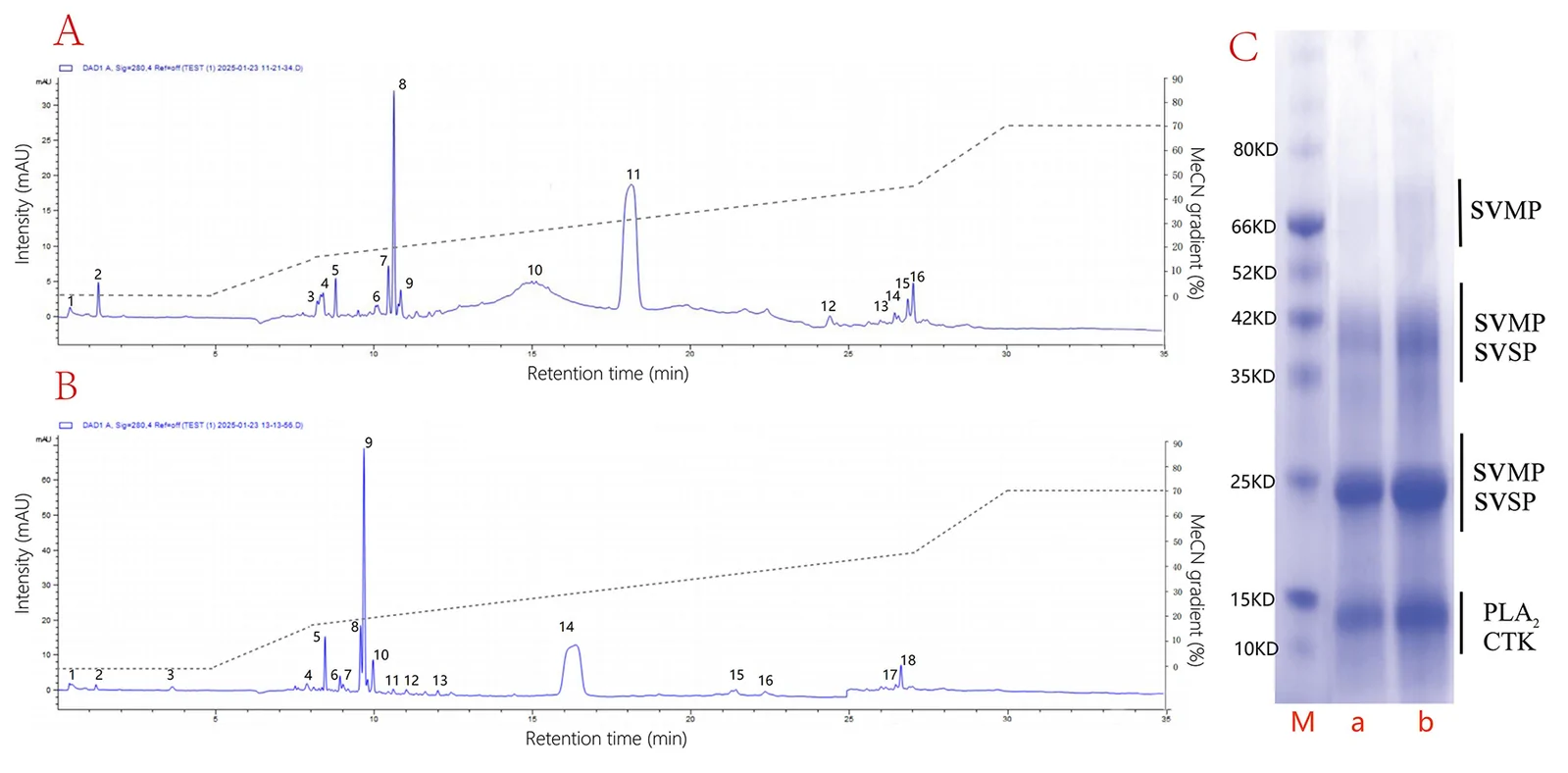
Integrated chromatographic and electrophoretic comparison of venoms from *G. brevicaudus* and *D. acutus*. **(A)** RP-HPLC chromatogram of *G. brevicaudus* venom; **(B)** RP-HPLC chromatogram of *D. acutus* venom; **(C)** SDS-PAGE profile of crude venoms (M: molecular weight marker; a: *G. brevicaudus* venom; b: *D. acutus* venom). RP-HPLC profiles reflect the overall compositional complexity of the venoms. Chromatographic profiles reflect overall compositional complexity, while the SDS-PAGE gel displays protein distribution across a 10-80 kDa range.

RP-HPLC chromatograms of *G. brevicaudus* (Figure 2A ) and *D. acutus* (Figure 2B ) exhibited complex multi-peak profiles, with all peaks eluting within approximately 28 min. The total number of peaks was comparable (16 vs 18), indicating similar compositional complexity. Both venoms displayed early peaks around 1 min, a dense cluster near 10 min, additional peaks between 15-20 min, and peaks in the 25-28 min range.

Despite these similarities, region-specific differences were observed. For example, *G. brevicaudus* exhibited more peaks than *D. acutus* in the 25-28 min interval. A peak at ~4 min was present only in *D. acutus*, whereas a peak around 15 min was detected only in *G. brevicaudus*.

*SDS-PAGE comparison of venom protein patterns*

SDS-PAGE analysis further supported these observations (Figure 2C ). Both *G. brevicaudus* (lane a) and *D. acutus* (lane b) venoms were loaded at equal amounts (20 µg per lane) and exhibited multiple protein bands spanning a wide molecular weight range (approximately 10-80 kDa). The overall banding patterns were highly similar between the two venoms.

Consistent with this observation, discrete protein signals were distributed across four major molecular weight regions (65-75 kDa, 40-50 kDa, 20-30 kDa, and 10-15 kDa), which correspond well to the typical size ranges of major *viper* venom toxin families reported in previous studies [19, 20].

Notwithstanding these similarities, apparent differences in band intensity were observed across several molecular weight regions. Under identical loading conditions, *G. brevicaudus* venom generally displayed lower staining intensity than *D. acutus* venom within corresponding molecular weight ranges. In addition, slight variations in band sharpness and density were noted in specific regions. However, these differences were primarily quantitative rather than qualitative, as no major differences in band presence or absence were observed.

*Integrated compositional comparison of the two venoms*

Overall, the strong concordance observed across proteomic, chromatographic, and electrophoretic analyses indicates that *G. brevicaudus* and *D. acutus* venoms possess highly conserved and broadly comparable compositional architectures. The observed interspecific differences were predominantly quantitative, reflecting variations in relative toxin abundance rather than major qualitative differences in toxin family composition.

### Comparable cytotoxic effects across multiple mammalian cell lines

To evaluate whether the conserved venom proteomes translated into similar cellular effects, cytotoxicity assays were conducted using MDCK II, RAW 264.7, and HEK 293T cells.

**Figure 3.**
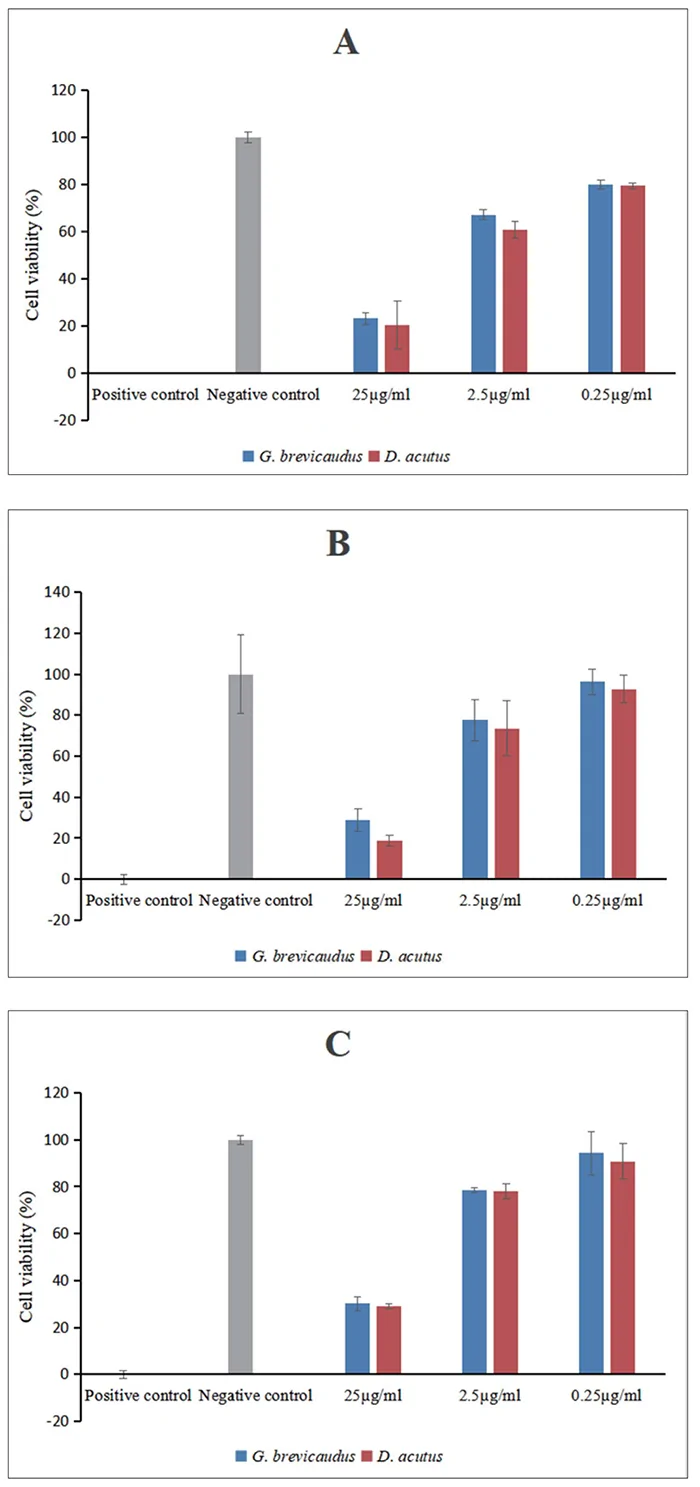
Concentration-dependent cytotoxic effects of *G. brevicaudus* and *D. acutus* venoms on cell viability. Viability profiles of **(A)** MDCK II, **(B)** RAW 264.7, and **(C)** HEK 293T cells after 48 h of exposure to venoms (0.25, 2.5, and 25 µg/mL). No statistically significant differences were observed between species at any concentration (*p* > 0.05). Data are mean ± SD (n = 3).

Both venoms exhibited clear concentration-dependent cytotoxicity across all three cell lines (Figure 3). At the highest concentration tested (25 μg/mL), cell viability was reduced to approximately 20-30% in all cell types, whereas lower concentrations resulted in progressively higher viability.

At corresponding concentrations, no statistically significant differences were observed between *G. brevicaudus* and *D. acutus* venoms in any of the tested cell lines (*p* > 0.05). These results indicate that the two venoms exert comparable cytotoxic effects, consistent with their shared abundance of cytotoxic and proteolytic toxin families (Figure 3).

### Blood-related enzymatic activities exhibit parallel functional profiles

Given that envenomation by both species is clinically characterized by coagulopathy and hemorrhagic manifestations, we next compared protease-related enzymatic activities.

*Protease and PLA₂ activities*

**Figure 4.**
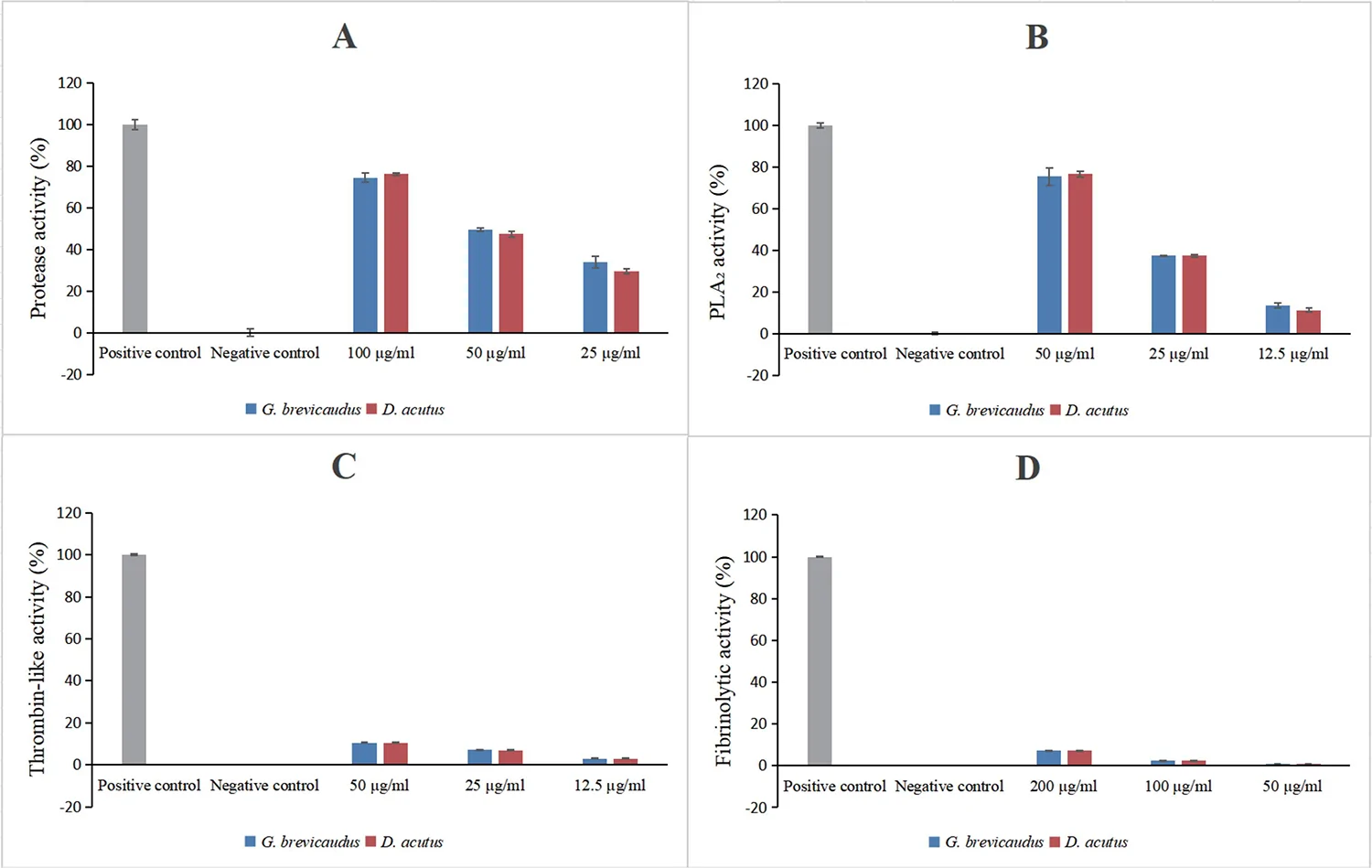
Parallel enzymatic activity profiles of *G. brevicaudus* and *D. acutus* venoms. **(A)** Total protease activity, **(B)** PLA₂ activity, **(C)** thrombin-like activity, and **(D)** fibrinolytic activity across various venom concentrations. No statistically significant differences were detected between the two species at any concentration (*p* > 0.05). Data represent mean ± SD (n = 3).

Protease activity assays revealed that both venoms displayed strong, concentration-dependent enzymatic activity, reaching approximately 75% of the positive control at 100 μg/mL. No significant differences were detected between the two venoms at any tested concentration (*p* > 0.05; Figure 4A ).

Similarly, PLA₂ activity assays demonstrated nearly identical activity profiles for both venoms, with a clear dose-dependent decline and no statistically significant interspecific differences (Figure 4B).

*Thrombin-like and fibrinolytic activities*

Both venoms exhibited measurable thrombin-like activity, increasing with venom concentration and reaching comparable maximal values. No significant differences were observed between *G. brevicaudus* and *D. acutus* venoms across all concentrations tested (*p* > 0.05; Figure 4C ).

Fibrinolytic activity assays revealed similarly low but concentration-dependent activity in both venoms, again with no statistically significant differences (Figure 4D ). Together, these findings demonstrate that the major hemostatic-disrupting enzymatic functions of the two venoms are functionally conserved.

### Low and comparable hemolytic activity across multiple erythrocyte models

To further assess membrane-disruptive effects, hemolytic activity was evaluated using murine, avian, ovine and human erythrocytes.

**Figure 5.**
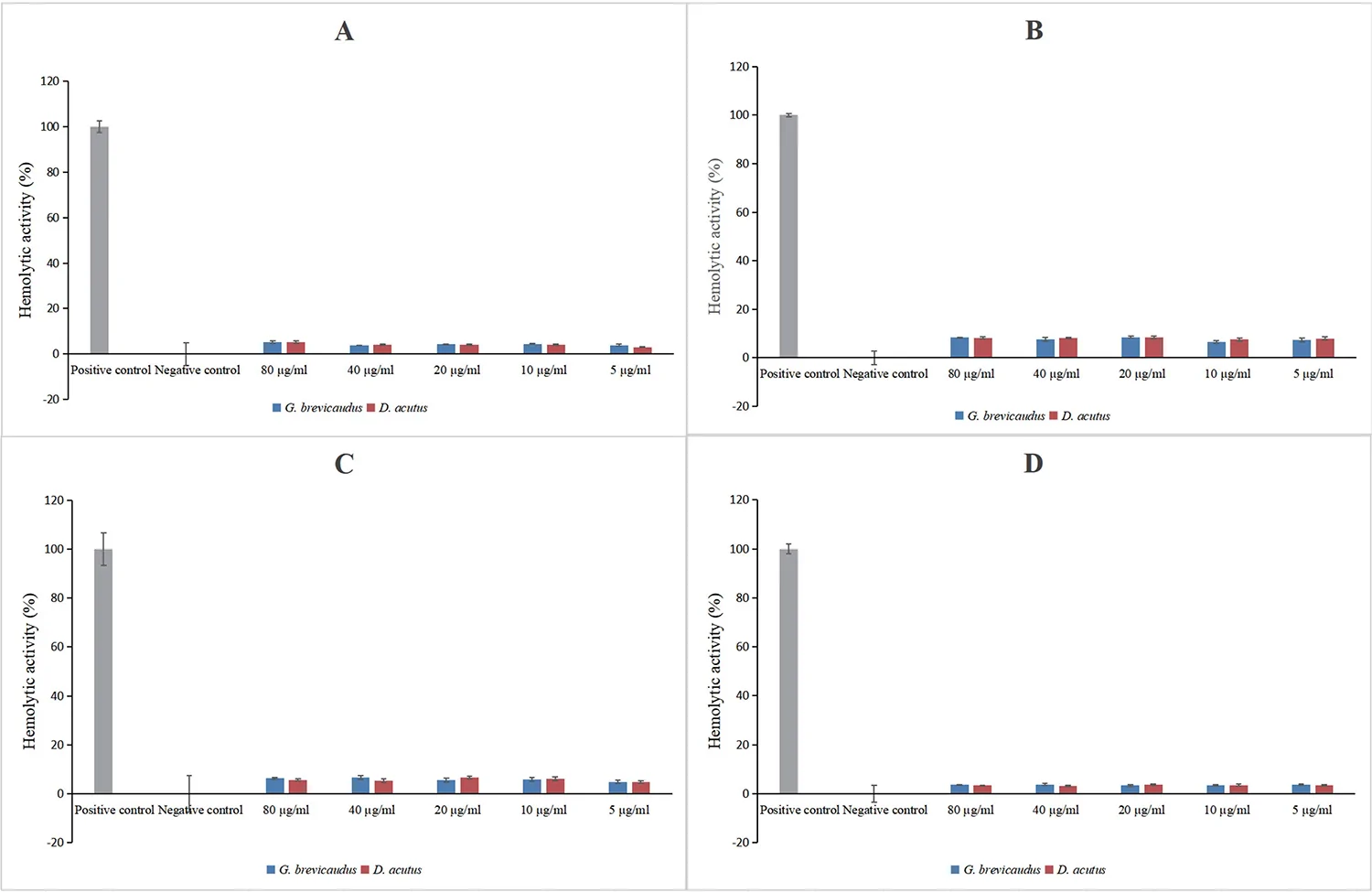
Hemolytic activity of *G. brevicaudus* and *D. acutus* venoms across multiple vertebrate erythrocyte models. Evaluation against **(A)** murine, **(B)** avian, **(C)** ovine, and **(D)** human erythrocytes at concentrations from 5 to 80 µg/mL. Both venoms induced minimal hemolysis (< 8%) with no significant differences between species (*p* > 0.05). Data represent mean ± SD (n = 3).

Across all tested concentrations and erythrocyte types, both venoms induced only mild hemolysis, generally below 8%. No statistically significant differences were observed between *G. brevicaudus* and *D. acutus* venoms in any erythrocyte model according to Student’s t-test analysis (*p* > 0.05; Figure 5).

These results indicate that direct erythrocyte lysis is not a dominant toxic mechanism for either venom, consistent with their classification as predominantly hemotoxic rather than hemolytic venoms.

### Limited effects on Ca²⁺ signaling and nitric oxide production

To examine potential effects on intracellular signaling and immune modulation, Ca²⁺ flux and nitric oxide (NO) production assays were performed.

**Figure 6.**
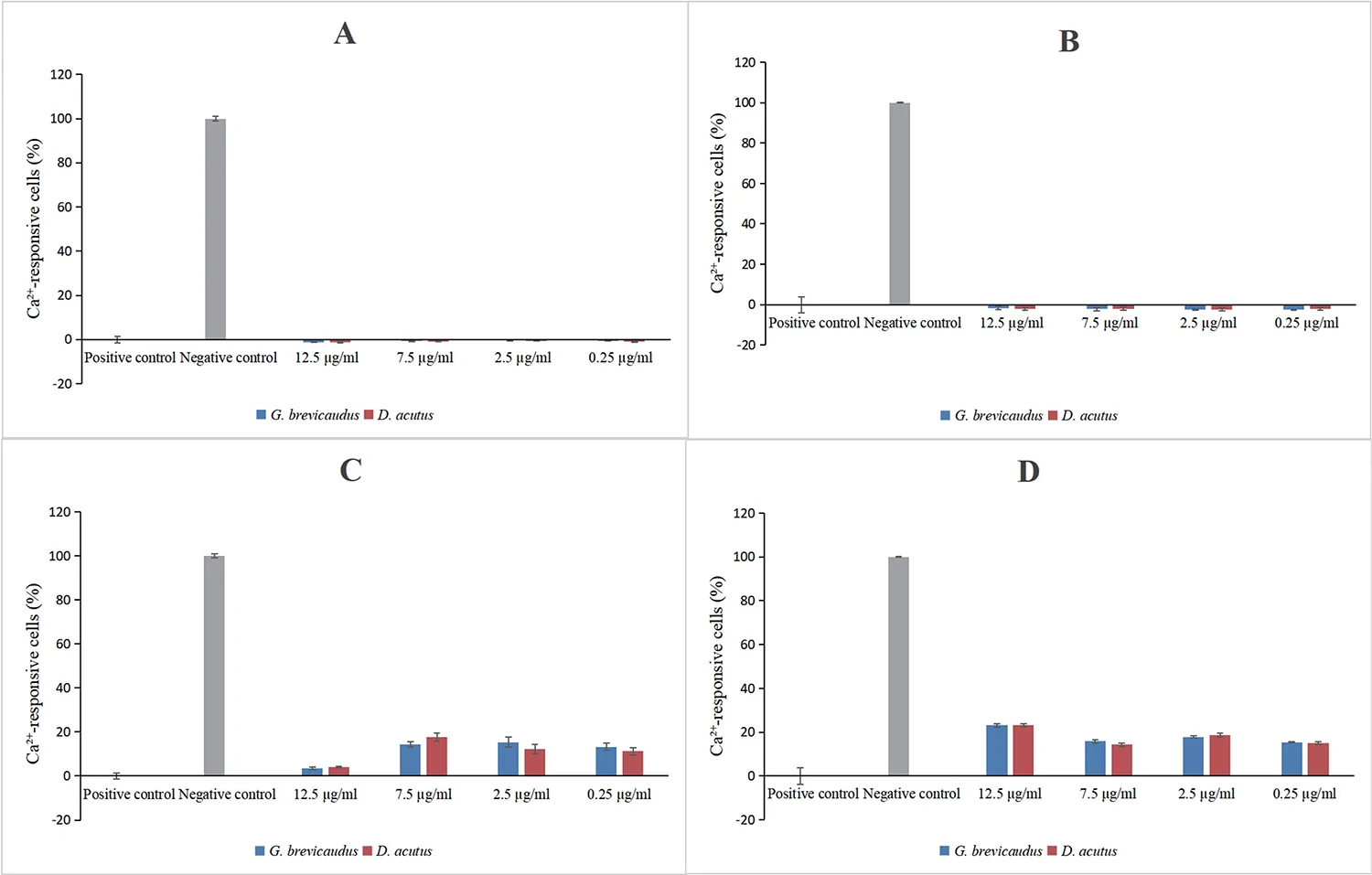
Intracellular Ca²⁺ responses in HEK 293T and MDCK II cells exposed to *G. brevicaudus* and *D. acutus* venoms. **(A, B)** Ca²⁺ induction and **(C, D)** Ca²⁺ inhibition dynamics across venom concentrations (0.25 to 12.5 µg/mL). Interspecific variations were not statistically significant (*p* > 0.05). Data represent mean ± SD (n = 3).

Both venoms induced only minimal changes in intracellular Ca²⁺ levels in HEK 293T and MDCK II cells under both induction and inhibition conditions. The observed effects were small, variable, and not significantly different between the two venoms at any concentration (*p* > 0.05; Figure 6).

**Figure 7.**
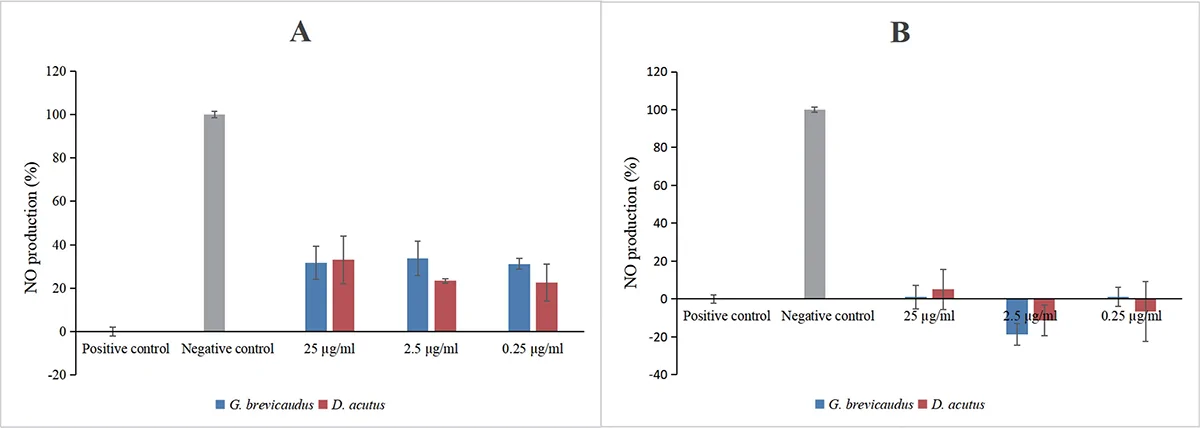
Modest regulatory effects of *G. brevicaudus* and *D. acutus* venoms on nitric oxide (NO) production in RAW 264.7 cells. **(A)** NO induction and **(B)** NO inhibition profiles at venom concentrations of 0.25, 2.5, and 25 µg/mL. No significant interspecific differences were observed (*p* > 0.05). Data represent mean ± SD (n = 3).

Similarly, neither venom markedly stimulated NO production in RAW 264.7 macrophages. Instead, both venoms exhibited modest inhibitory effects on NO synthesis, with comparable inhibition levels and no statistically significant interspecific differences (*p* > 0.05; Figure 7).

These findings suggest that Ca²⁺-mediated signaling and NO-related inflammatory modulation are not primary targets of either venom under the tested conditions.

## Discussion

Snake venom composition is influenced by factors such as phylogenetic relationships, ecological adaptation, and prey preference [26-29]. *G. brevicaudus* and *D. acutus* both belong to the family Viperidae but are classified into the distinct genera (*Gloydius* and *Deinagkistrodon*, respectively), reflecting divergent evolutionary histories [1, 2]. However, despite taxonomic divergence among viperid genera, their venoms often retain a relatively conserved repertoire of functionally important toxin families, leading to similar hemotoxic manifestations [30, 31]. Here, we used a unified experimental platform to systematically compare the proteomic profiles and biological activities of these two medically important venoms to determine whether distinct genera share a conserved hemotoxic architecture.

Proteomic analysis revealed a substantial overlap between *G. brevicaudus* and *D. acutus* venoms, with 147 shared protein entries. In both venoms, SVMP, SVSP, PLA₂, and CTLP were the dominant toxin families, although their protein entry numbers and relative abundances varied. These findings align with previous proteomic studies of *D. acutus* [16-19, 31, 32] and *G. brevicaudus* [20], which demonstrated that these four families constitute the major hemotoxic components of viper venoms.

Chromatographic and electrophoretic profiling further corroborated these observations, confirming broadly comparable compositional architectures between the two venoms. The observed molecular weight distribution patterns match the typical sizes of SVMP, SVSP, PLA₂, and CTLP families [19, 20]. Notably, variations in peak distribution and band intensity likely reflect species-specific quantitative differences in toxin abundance rather than major qualitative divergence.

Several low-abundance toxin families (CRISP, PDE, PLB, 5′-NT, NGF, and 3FTx) were detected in both venoms, while LAAO occurred exclusively in *G. brevicaudus*. Although previous studies reported LAAO in *D. acutus* [16,31,32], it was undetected here. Conversely, 3FTx, PDE, and PLB are reported for the first time in *G. brevicaudus*, expanding upon known profiles [20, 31, 33]. Interestingly, the neurotoxic 3FTx family was 4.5-fold more abundant in *G. brevicaudus* (0.77%) than in *D. acutus* (0.17%), which may link to the neurological manifestations associated with *G. brevicaudus* envenomation [13, 14]. Additionally, the hemotoxic components PDE and PLB are linked to anticoagulant and hemolytic activities, respectively [34-36]. Discrepancies with literature likely stem from variations in venom sources, geography, mass spectrometry platforms, databases, or bioinformatics workflows.

Proteomic similarities reflected the hemotoxic profiles of both venoms, aligning with trends reported for *D. acutus* [19]. Both venoms exhibited strong, concentration-dependent proteolytic and PLA₂ activities, while fibrinolytic and thrombin-like activities were weaker. This pattern contrasts with the prominent clinical coagulopathy and tissue necrosis, suggesting that pathology is mediated via synergistic toxin interactions rather than isolated components [37]. Consequently, venom pathogenicity reflects integrated interaction networks: SVMPS degrade vascular basement membranes to cause local hemorrhage [38]; SVSPS drive coagulopathy via fibrinogen cleavage [39]; CTLP impair hemostasis by binding platelet membrane glycoproteins (e.g., agkisacutacin from *D. acutus* inhibiting platelet adhesion via GPIbα [40]); and PLA₂s hydrolyze phospholipids, releasing inflammatory mediators that exacerbate tissue injury [41]. Together, these coordinated mechanisms drive envenomation pathology in both species.

Hemolytic assays across human, murine, ovine, and avian erythrocytes revealed low activity. The low abundance of PLB (1.76% in *G. brevicaudus* and 3.62% in *D. acutus*) likely accounts for this weak effect. Conversely, both venoms showed marked, concentration-dependent cytotoxicity in all three cell lines, yet neither significantly altered intracellular Ca²⁺ release in HEK 293T or MDCK II cells or NO production in RAW 264.7 cells, mirroring patterns from Avella et al. [19]. These findings confirm that the toxic effects are primarily hemotoxic, while direct impacts on cellular signaling and immunomodulation are limited.

Despite proteomic and functional similarities, the LD_50_ of *G. brevicaudus* venom (0.49 mg/kg) is significantly lower than that of *D. acutus* (4.4 mg/kg), showing higher potency per unit [15]. This divergence may stem from the greater complexity of *G. brevicaudus* (259 protein entries vs. 197 in *D. acutus*). As shown by Pucca et al. [42] and Rudresha et al. [43], synergistic interactions within complex toxin networks can produce higher toxicity than individual components alone. Thus, venom lethality may depend heavily on network complexity and synergy involving low-abundance components.

The predominance of hemotoxic families suggests a conserved functional strategy among East Asian viperid snakes to rapidly disrupt prey vascular and tissue integrity [29, 44, 45]. Evolutionarily, the observed variations likely reflect adaptation to distinct ecological pressures. *G. brevicaudus*, possessing a smaller body size and lower venom yield, may compensate by increasing venom potency to maintain predatory efficiency. In contrast, *D. acutus* relies on high venom output for prey immobilization. This divergence between a “high-potency/low-yield” strategy and a “high-yield/moderate-potency” strategy underlies the differences in complexity and toxicity between the two species.

Clinically, this conserved hemotoxic architecture explains the overlapping hemorrhagic and coagulopathic manifestations of both envenomations. Although species-specific antivenoms are used in China, limitations regarding specificity, adverse reactions, and accessibility persist [46-48]. Consequently, monoclonal antibodies targeting SVMP, SVSP, PLA₂, and 3FTx offer promising avenues for next-generation, broad-spectrum therapies [49-51]. Furthermore, small-molecule inhibitors show immense potential: the metal chelator DMPS and metalloproteinase inhibitor marimastat (alone or with varespladib) neutralize dermonecrosis [52-54], while the PLA₂ inhibitor varespladib has shown broad preclinical efficacy and positive phase II clinical results [55-57]. This shared architectural framework highlights viable targets for universal therapeutic strategies.

Although individual RP-HPLC fractions were not directly characterized via fraction-specific proteomic workflows, their elution profiles mirror well-documented chromatographic behaviors for viperids [20, 58, 59]. Typically, small peptides elute early, intermediate proteins (PLA₂, SVSP, CRISP, and CTLP) elute in mid-fractions, and larger components (SVMP and LAAO) elute late, as demonstrated in *Bothrops atrox* [58], *Echis carinatus* [59], and *G. brevicaudus* [20]. Thus, the dense cluster near 10 min likely represents intermediate-weight toxins, while the 25-28 min peaks match SVMP-rich fractions. While the lack of direct fraction-resolved proteomics prevents definitive peak assignment, future peak-by-peak characterization and in vivo functional validation will clarify individual fractional roles and synergism.

## Conclusions

By integrating comparative proteomics with functional toxicological assays, this study demonstrates that *G. brevicaudus* and *D. acutus* venoms share highly conserved hemotoxic and cytotoxic profiles. This functional alignment provides a mechanistic explanation for their clinically overlapping envenomation syndromes. Ultimately, these findings emphasize the importance of evaluating shared toxicological mechanisms when interpreting snakebite pathology, while establishing a framework for future investigations into antivenom cross-reactivity and broad-spectrum therapeutic development.

### Abbreviations

3FTx: three-finger toxin; 5′-NT: 5′-nucleotidase; CRISP: cysteine-rich secretory proteins; CTLP: C-type lectin-like proteins; LAAO: L-amino acid oxidase; NGF: nerve growth factor; PDE: phosphodiesterase; PLA₂: phospholipase A₂; PLB: phospholipase B; RP-HPLC: reverse-phase high-performance liquid chromatography; SVMP: snake venom metalloproteinases (peptidase M1 and M14 families); SVSP: snake venom serine proteases (peptidase S1, S9B, and S10 families). 

## Availability of data and materials

 The original contributions presented in this study are included within the article and its supplementary files.

## Supplementary material

The following online material is available for this article:

Additional file 1. Complete list of all protein entries identified in the venoms. Detailed information on protein identification and annotation was obtained through MaxQuant analysis, with component IDs assigned based on search results from the UniProt database. Red shading indicates protein components common to both venoms. **(A)** Complete protein entries identified in the venom of *Gloydius brevicaudus*. **(B)** Complete protein entries identified in the venom of *Deinagkistrodon acutus*.

Additional file 2. Relative abundance (%) of venom protein families identified in *G. brevicaudus* and *D. acutus* based on normalized abundance values obtained from MaxQuant analysis. 3FTx: three-finger toxin; 5′-NT: 5′-nucleotidase; CRISP: cysteine-rich secretory protein; CTLP: C-type lectin-like protein; LAAO: L-amino acid oxidase; NGF: nerve growth factor; PDE: phosphodiesterase; PLA₂: phospholipase A₂; PLB: phospholipase B; SVMP: snake venom metalloproteinase; SVSP: snake venom serine protease.
